# Outcomes of Salvage Trabeculectomy in Japanese Patients with Open-Angle Glaucoma and Persistent Intraocular Pressure Elevation Following Trabectome or Microhook Ab Interno Trabeculotomy

**DOI:** 10.3390/jcm15124826

**Published:** 2026-06-21

**Authors:** Toshiki Oka, Mari Sakamoto, Sotaro Mori, Kaori Ueda, Yuko Yamada-Nakanishi, Makoto Nakamura

**Affiliations:** Division of Ophthalmology, Department of Surgery, Kobe University Graduate School of Medicine, Hyogo 650-0017, Japan; oka7716@med.kobe-u.ac.jp (T.O.); smori@med.kobe-u.ac.jp (S.M.); kueda@med.kobe-u.ac.jp (K.U.); yamaday@med.kobe-u.ac.jp (Y.Y.-N.); manakamu@med.kobe-u.ac.jp (M.N.)

**Keywords:** trabeculectomy, Trabectome, microhook ab interno trabeculotomy, glaucoma

## Abstract

**Background/Objectives:** The objective was to describe the one-year outcomes of salvage trabeculectomy (TLE) in eyes with persistent elevation of intraocular pressure (IOP) requiring early surgical intervention after failed minimally invasive glaucoma surgery (MIGS). **Methods**: This retrospective observational study included 38 eyes of 38 consecutive Japanese patients who underwent TLE within 100 days after Trabectome (TOM) or microhook ab interno trabeculotomy (μTLO) because of uncontrolled IOP despite maximally tolerated medical therapy. Surgical success was defined as (1) IOP reduction ≥30% from baseline, (2) 5 < IOP < 18 mmHg, (3) no additional glaucoma surgery, and (4) no loss of light perception. The Kaplan–Meier method was used to estimate the one-year success rate. Changes in IOP, medication use, best-corrected visual acuity (BCVA), and mean deviation (MD) were analyzed using the Wilcoxon matched-pairs signed-rank test and a linear mixed-effects model. **Results**: The median interval between MIGS and TLE was 41.5 days (interquartile range, 28–70 days). The one-year surgical success rate was 86.8% (Kaplan–Meier estimate). IOP and medication use were significantly reduced after TLE (*p* < 0.0001) and remained stable throughout the 12-month follow-up. BCVA did not differ significantly between baseline and 12 months after TLE, whereas a small but statistically significant difference in MD was observed. No serious vision-threatening complications were encountered. **Conclusions**: TLE performed shortly after failed MIGS achieved substantial IOP reduction with acceptable safety over a one-year follow-up period. TLE may be considered as one of the surgical options in cases where sufficient IOP reduction cannot be achieved after failed MIGS, and no effective alternative treatments are available.

## 1. Introduction

Minimally invasive glaucoma surgery (MIGS) has become an increasingly popular option for early- to moderate-stage glaucoma due to its favorable safety profile. Ab interno procedures such as Trabectome (TOM) and microhook trabeculotomy (μTLO) are widely used in Japan to enhance aqueous outflow through Schlemm’s canal [1]. However, the reported 1-year success rate of MIGS has ranged from 40% to 70% [2,3,4,5,6,7], reflecting heterogeneity in study populations and success definitions, including complete and qualified success. A subset of patients requires additional glaucoma surgery within the first year. For example, Kono et al. reported that 26% of eyes required further surgery within one year after TOM [6], while Tanito et al. and Okuda et al. reported rates of 8.1% and 20% following μTLO, respectively [3,7]. These studies provide important insights into the overall failure rates of MIGS but do not specify the timing of reoperation. In clinical practice, we encounter patients who require reoperation very early—within weeks to a few months—after MIGS due to persistent high intraocular pressure (IOP) despite maximally tolerated medical therapy. Although the exact proportion of such early failures remains unclear, they represent a clinically important subgroup that warrants further investigation.

Trabeculectomy (TLE) is often performed as an additional procedure in such cases. Because TOM and μTLO do not involve conjunctival dissection, TLE can theoretically be performed shortly after MIGS. However, concurrent cataract surgery, which is frequently performed with MIGS, is a known risk factor for bleb failure due to postoperative inflammation [8,9,10,11]. Moreover, μTLO has been reported to be associated with postoperative anterior chamber flare compared with some other glaucoma procedures [12], although the clinical significance of this finding in the setting of subsequent filtering surgery remains unclear.

Although one study has reported that failed TOM does not adversely affect subsequent TLE outcomes [13], no prior studies have specifically evaluated the safety and efficacy of TLE performed shortly after failed MIGS in Japanese patients. Such cases represent a clinically challenging scenario in which IOP remains persistently elevated despite maximally tolerated medical therapy, leaving limited treatment options and necessitating surgical intervention as a salvage strategy. These situations are relatively uncommon but represent an important unmet clinical need in glaucoma management.

This study aims to describe the one-year outcomes of TLE in a highly selected population of patients who required early TLE due to uncontrolled IOP following MIGS.

## 2. Materials and Methods

This was a retrospective observational study conducted at a single institution. The Institutional Review Board of Kobe University Hospital approved the study protocol (approval no. B230084), and the study was conducted in accordance with the tenets of the Declaration of Helsinki. Details of this study were posted on our hospital’s website and on notices within the hospital, and participants were given the opportunity to opt out. Considering the retrospective nature of the study and the use of fully anonymized data, the Institutional Review Board of Kobe University Hospital waived the requirement for obtaining informed consent from individual participants. Throughout the study period, participants’ privacy rights were strictly protected.

We reviewed the medical records of consecutive glaucoma patients aged ≥18 years who underwent TLE with mitomycin C between January 2015 and March 2022. The study group included patients who underwent TLE within 100 days after failed TOM or μTLO due to persistent IOP elevation despite maximally tolerated medical therapy. There was no predefined quantitative threshold of IOP or number of medications to declare MIGS failure. In clinical practice, the decision to proceed to TLE was based on a comprehensive assessment of postoperative IOP control and disease severity. Specifically, TLE was considered when eyes demonstrated persistently elevated IOP despite maximally tolerated medical therapy, inability to intensify topical therapy because of adverse effects or medication intolerance, and/or advanced glaucomatous damage in which continued elevation of IOP was judged to pose a substantial risk of further visual field loss.

Those who underwent TLE with amniotic membrane transplantation, or those with less than 12 months of follow-up were excluded. A one-eye-per-patient design was pre-specified. When both eyes met the inclusion criteria, only the first operated eye was included to avoid inter-eye correlation.

We collected data on age, sex, glaucoma type, prior ocular surgeries, concurrent cataract surgery, intra- and postoperative complications, best-corrected visual acuity (BCVA) in a logarithm of the minimum angle of resolution (logMAR), mean deviation (MD) from Humphrey visual field testing, IOP (Goldmann applanation tonometry), number of glaucoma medications, and additional glaucoma surgeries. The combination of eye drops and oral acetazolamide was counted as two medications.

The primary outcome was the 1-year surgical success rate of TLE, defined as meeting all of the following criteria: (1) IOP reduction ≥30% from baseline, (2) 5 < IOP < 18 mmHg, (3) no additional glaucoma surgery, and (4) no loss of light perception. Regarding IOP, failure was defined as deviation from these criteria on two consecutive visits after TLE with or without the use of glaucoma medications.

At our institution, small incisional bleb revision is routinely performed instead of bleb needling for early postoperative bleb scarring, as described in our previous report [14]. Therefore, bleb needling or revision was not considered an additional glaucoma surgery in the primary outcome definition. However, as a sensitivity analysis, surgical success was additionally evaluated using a stricter World Glaucoma Association (WGA) conformant definition, in which bleb needling or revision procedures were counted as surgical failure.

Secondary outcomes included changes in IOP, medication use, BCVA, MD, and surgical complications. Hyphema was graded using the Shimane University RLC (R, red blood cells; L, layered hyphema; and C, clots) scoring system (SU-RLC) [15]. In SU-RLC, layered hyphema was classified into four categories: 0, no layer formation; 1, layer formation < 1 mm; 2, layer formation below the inferior pupillary margin; and 3, layer formation above the inferior pupillary margin. Layered hyphema was assessed based on slit-lamp examination and anterior segment photographs obtained on postoperative day 1 or 2.

Because this was a retrospective observational study, no a priori sample size or power calculation was performed, and all analyses should be interpreted as descriptive and exploratory.

Statistical analyses were performed using the Wilcoxon matched-pairs signed-rank test and a mixed-effects model. For longitudinal IOP analysis, a linear mixed-effects model was used with time as a fixed effect and eye as a random intercept to account for within-eye correlation. An autoregressive covariance structure (AR [1]) was assumed for repeated measures, reflecting the assumption that measurements closer in time are more strongly correlated. All statistical analyses were performed using MedCalc Statistical Software version 20.010 (MedCalc Software Ltd., Ostend, Belgium) and IBM SPSS Statistics for Windows, Version 32.0 (IBM Corp., Armonk, NY, USA) with type I error for significance set at *p* < 0.05.

Microsoft 365 Copilot, version available as of June 2026, was used solely for language editing and clarity improvement during manuscript preparation. It was not used for study design, data analysis, or interpretation of results, and all AI-assisted outputs were reviewed and edited by the authors.

### Surgical Procedures

Details of the surgical procedures for TOM/µTLO have been published elsewhere [6,7,16]. Briefly, the aqueous humor was replaced with a viscoelastic material through a corneal side port. The patient’s head and the surgical microscope were adjusted to optimize visualization of the anterior chamber angle using a gonioprism lens. A Trabectome probe or Tanito ab interno trabeculotomy microhook was inserted through the side port, and the inner wall of the trabecular meshwork was dissected or incised, respectively. The viscoelastic material was removed by irrigation through the side port. When cataract surgery was combined, it was performed either before TOM/µTLO or after a continuous curvilinear capsulorrhexis followed by TOM/µTLO and then the rest of the cataract surgery procedure.

In all cases, the subsequent TLE was performed with fornix-based incision at superior conjunctiva. After administering topical anesthesia (2% lidocaine) to the sub-Tenon space, a clear cornea stay suture (6-0 vicryl) was placed to rotate the eye downwards to expose the superior conjunctiva. After subconjunctival injection of 2% lidocaine, a conjunctival incision was made, and the Tenon tissue was dissected to expose the sclera. After diathermy hemostasis, a rectangular scleral flap of 4 mm × 4 mm with its base at the corneal limbus was created with a thickness of half a layer of sclera. Sponges soaked in 0.04% mitomycin C were applied for 3 min, then the surgical site was washed with balanced salt solution. A paracentesis is created in the peripheral cornea, and a second scleral flap of the remaining half thickness of sclera was created and dissected anteriorly until the Schlemm’s canal was identified. A blade was inserted into the anterior chamber from both anterior corners of the second flap, and the second flap was excised along with trabecular block using Vannas scissors. The first scleral flap was sutured with 10-0 nylon. Finally, the conjunctiva was sutured with 10-0 nylon. Postoperative topical therapy consisted of betamethasone and antibiotic eye drops four to six times a day depending on the bleb status and IOP, then tapered thereafter.

## 3. Results

A total of 39 eyes from 39 patients were initially identified as the study group (TLE after failed MIGS), as illustrated in Supplementary Figure S1. One eye with chronic angle-closure glaucoma was excluded from the analysis to ensure a more homogeneous study population. Therefore, 38 eyes from 38 patients were included in the final analysis. During the study period, approximately 885 MIGS procedures were performed at our institution. Although this number is approximate and includes both eyes, it provides a general indication of the rarity of early surgical conversion to TLE.

The median interval between MIGS and TLE in the study group was 41.5 days (interquartile range, 28–70 days). Among the study group, 9 eyes had undergone TOM and 29 had undergone μTLO. Thirteen eyes (34%) had undergone concurrent cataract surgery at the time of MIGS.

Table 1 summarizes the baseline demographic and clinical characteristics of the study group. The cohort comprised a highly selected population characterized by substantial IOP elevation at baseline and a high prevalence of secondary glaucoma with frequent prior ocular surgery. Baseline IOP and medication burden were high at the time of TLE, reflecting the refractory nature of IOP elevation in this study population.

**Figure 1:**
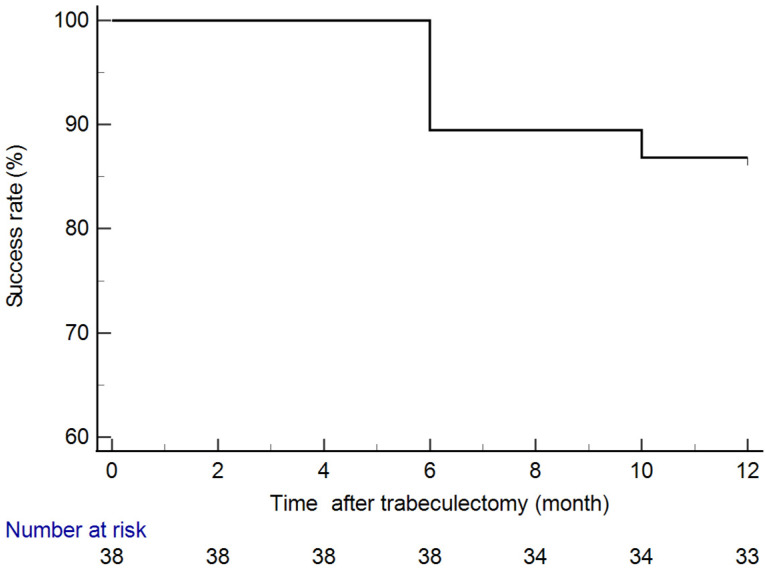
Kaplan–Meier curve for surgical success rate up to 12 months after trabeculectomy. Censored observations represent eyes without surgical failure at the last follow-up. The number of eyes at risk at each time point is shown below the plot. Because this figure depicts a single study group, a log-rank test was not performed.

At 1 year post-TLE, the surgical success rate in the study group was 86.8% (95% CI, 76.1–97.5) (Figure 1). The Kaplan–Meier estimated success rates were 100% (95% CI, 100–100) at 3 months and 89.5% (95% CI, 79.7–99.3) at 6 months. In an exploratory analysis stratified by the interval between MIGS and TLE (≤30, 31–60, and 61–100 days), no significant difference in surgical success was observed among groups (log-rank test, *p* = 0.71; Supplementary Figure S2). When surgical success was redefined using a stricter, WGA-conformant criterion that counted bleb needling/revision as failure, the Kaplan–Meier estimated success rate decreased, as shown in Supplementary Figure S3. Similarly, surgical success did not differ significantly according to the presence or absence of concomitant cataract surgery at the time of MIGS (log-rank test, *p* = 0.15; Supplementary Figure S4).

**Figure 2:**
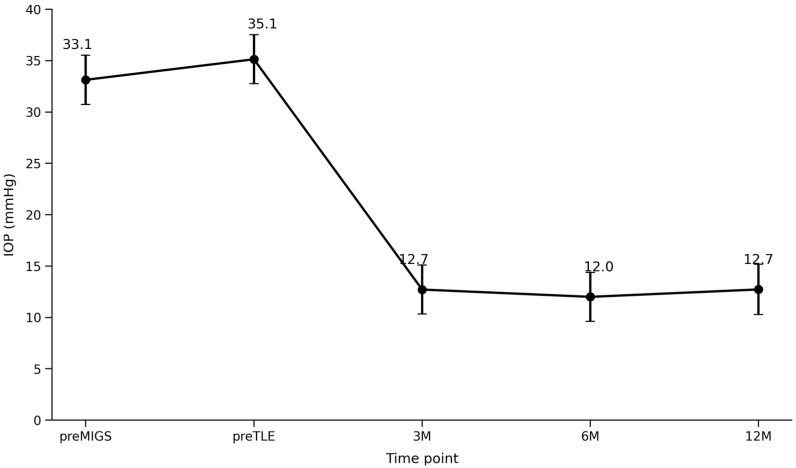
Changes in intraocular pressure over time. Values represent estimated marginal means with 95% confidence intervals derived from a linear mixed-effects model. Data were available from 38 eyes at pre-MIGS, pre-TLE, 3 months, and 6 months, and from 37 eyes at 12 months. IOP, intraocular pressure; MIGS, minimally invasive glaucoma surgery; TLE, trabeculectomy.

Changes in IOP over time in the study group are shown in Figure 2. Estimated marginal means derived from a linear mixed-effects model demonstrated a marked reduction in IOP after TLE, which was maintained throughout the 12-month follow-up period.

Changes in ophthalmic parameters before and after TLE in the study group are shown in Table 2. IOP and medication scores showed a significant reduction at 12 months after TLE compared with both the pre-MIGS and pre-TLE periods. BCVA did not differ significantly between the pre-TLE baseline and 12 months after TLE, whereas a small but statistically significant difference in visual field MD was observed between the same time points. In addition, per-eye line plots illustrating changes between the pre-MIGS and pre-TLE periods show persistent IOP elevation and an increasing medication burden prior to TLE (Supplementary Figures S5 and S6).

Postoperative outcomes and complications at 12 months after TLE in the study group are summarized in Table 3. Surgical success was maintained in most eyes, and postoperative complications were limited.

## 4. Discussion

This study investigated the one-year outcomes of salvage TLE performed shortly after unsuccessful TOM/µTLO in a highly selected population with persistently elevated IOP. Our findings suggest that TLE achieved substantial IOP reduction and an acceptable surgical success rate over a 12-month follow-up period, even when performed early after failed MIGS. Although TLE after failed glaucoma surgery has been reported, the present study focuses on a specific clinical scenario involving early surgical intervention for persistent and medically uncontrolled IOP after MIGS, which has not been well characterized. Importantly, the elevated IOP observed in this cohort was not transient but persisted for several weeks despite maximally tolerated medical therapy, requiring surgical intervention.

The heterogeneity in baseline characteristics observed in this cohort reflects the real-world complexity of this salvage population. The clinical characteristics of eyes in which IOP remains uncontrolled after TOM/µTLO have not been well defined. In the present cohort, secondary glaucoma was common, and many eyes had a history of prior ocular surgery, both of which may contribute to insufficient IOP control after ab interno procedures. This distribution of glaucoma subtypes likely reflects the characteristics of this specific salvage population, in which eyes with more complex clinical backgrounds are more prone to early failure of MIGS, rather than representing a methodological bias. In addition, layered hyphema was frequently observed after the initial MIGS; however, most hemorrhages were mild to moderate, with SU-RLC scores of 2 or less. Further studies are required to clarify the mechanisms underlying persistent IOP elevation following TOM/µTLO. Although patients aged ≥ 18 years were eligible for inclusion, younger patients were rare in this cohort: only three patients were younger than 45 years, and only one eye had a possible diagnosis of juvenile glaucoma. This suggests that the markedly elevated preoperative IOP observed in the present study is unlikely to be attributable to the inclusion of juvenile glaucoma cases and instead reflects severe early failure of MIGS in a salvage population.

Despite the early timing of TLE after failed MIGS, surgical success was maintained in the majority of eyes throughout the one-year follow-up. Jea et al. previously reported a one-year success rate of 77.2% for TLE after failed TOM, with a longer interval between the two procedures. Although the interval between TOM/µTLO and TLE was shorter in our cohort, the overall postoperative course was consistent with previously reported experiences, suggesting that prior ab interno procedures do not necessarily preclude favorable outcomes of subsequent TLE. Although a history of TLE has been reported to be a risk factor for subsequent TLE failure [13,17], our results and those of Jea et al. suggest that unsuccessful TOM, which is considered ab interno TLE, may not adversely affect subsequent ab externo TLE outcomes. The fact that TOM is an ab interno procedure that does not violate the conjunctiva may be one possible explanation for the observed outcomes.

Visual acuity deterioration was observed at the time of TLE in approximately half of the eyes, likely due to corneal edema related to uncontrolled IOP and/or anterior chamber hemorrhage. At 12 months after TLE, visual acuity recovered to baseline levels in some eyes, whereas others remained deteriorated. This may be attributable to glaucomatous damage caused by prolonged IOP elevation prior to TLE and to surgically induced astigmatism. A small decline in visual field MD was also observed between baseline and 12 months after TLE. However, a 1.7 dB change over a single year, based on only two Humphrey visual field examinations, is within or near the expected test–retest variability for moderate-to-advanced visual field loss and cannot be distinguished from regression-to-the-mean, learning effects, or postoperative media changes. Accordingly, this finding should be regarded as hypothesis-generating and does not allow definitive conclusions regarding true visual field progression over this short interval.

An important consideration in interpreting these results is the highly selected nature of the study population. The majority of eyes in this cohort had secondary glaucoma, a history of prior ocular surgery, very high preoperative IOP, and a substantial medication burden, indicating a salvage population with refractory IOP elevation after MIGS. Therefore, the findings of this study should not be generalized to all cases of MIGS failure. It is possible that eyes with primary open-angle glaucoma and milder IOP elevation after MIGS may achieve acceptable pressure control without requiring early conversion to TLE, and the indications for TLE in such eyes may differ substantially from those in the present cohort. The current results primarily inform the role of TLE as a salvage procedure in heavily pretreated eyes, particularly in secondary glaucoma, rather than its routine use in less advanced or primary diseases. Notably, the favorable surgical outcomes observed in this challenging population suggest that prior canal-based MIGS and early postoperative inflammation do not necessarily preclude successful TLE. However, whether similar or superior outcomes can be expected in eyes with primary open-angle glaucoma and less severe MIGS failure remains uncertain and warrants further investigation.

This study has several limitations. Its retrospective nature and relatively small sample size limit the identification of factors associated with surgical failure. Decisions regarding the timing of TLE after failed MIGS were made at the discretion of individual surgeons, reflecting real-world clinical practice. These factors introduce inherent selection bias. Therefore, our findings should be interpreted within the context of a highly selected salvage population rather than as generalized evidence applicable to all cases of MIGS failure. Larger prospective studies are needed to further characterize this unique patient population and to optimize treatment strategies.

Another important limitation is that TLE comprises a variety of surgical techniques with institutional and surgeon-specific variations. Therefore, the outcomes observed in this study reflect the specific surgical method employed at our institution, and similar results should not be assumed for alternative TLE techniques or procedural modifications. In addition, detailed postoperative angle findings, including peripheral anterior synechiae, were not systematically or quantitatively assessed due to the retrospective study design, and their potential contribution to postoperative IOP elevation cannot be fully evaluated.

## 5. Conclusions

TLE effectively reduced persistently elevated IOP after failed TOM/µTLO, even when performed in the early postoperative period. Given the limited effectiveness of medical therapy in this setting, TLE may be considered as one of the surgical options when sufficient IOP reduction cannot be achieved after failed MIGS.

## Figures and Tables

**Table 1:** Baseline demographic and clinical characteristics of the study group.

	Study Group (n = 38)
Age (yrs, IQR)	66.5 (57 to 74) (range 21 to 89)
Sex (male, %)	25 (65.8)
Eye (Right eye, %)	17 (44.7)
MD (dB, IQR)	−11.9 (−21.0 to −7.0)
BCVA (logMAR, IQR)	0.05 (−0.02 to 0.43)
IOP (mmHg, IQR)	33.0 (28.0 to 42.0)
Medication score (IQR)	6.0 (4.0 to 6.0)
Type of glaucoma	
POAG (eye, %)	13 (34.2)
SOAG (eye, %)	25 (65.8)
PXG (eye)	14 (2 eyes with IOL donasis, 1 eye with atopic dermatitis)
Uveitis (eye)	4
Steroid (eye)	4 (3 eyes with atopic dermatitis, 1 eye with Myasthenia gravis)
Post-vitrectomy (eye)	2
Post-CRVO (eye)	1 (without neovascularization)
Previous operation	
None (eye, %)	20 (52.6)
Yes (eye, %)	18 (47.4)
	Cataract 7
	SLT 3
	Vitrectomy 2
	iStent 1
	TLO ab externo 4
	TLE 1
Intervals between TOM/μTLO and TLE (days, IQR)	41.5 (28 to 70) (range 8 to 98)
Type of MIGS	TOM 9, μTLO 29
Concurrent cataract surgery (eye, %)	13 (34)
Complication of prior TOM/μTLO (eye)	Posterior capsule rupture during concurrent cataract surgery 1
	Layered hyphema 18
	Uncontrolled IOP elevation 38

IQR, interquartile range; MD, mean deviation of Humphrey visual field; BCVA, best corrected visual acuity; logMAR, logarithm of the minimum angle of resolution; IOP, intraocular pressure; POAG, primary open-angle glaucoma; SOAG, secondary open-angle glaucoma; PXG, pseudoexfoliative glaucoma; IOL, intraocular lens; CRVO, central retinal vein occlusion; SLT, selective laser trabeculoplasty; TLO, trabeculotomy; TLE, trabeculectomy; TOM, trabectome; μTLO, microhook ab interno TLO. Layered hyphema was assessed based on slit-lamp examination and anterior segment photographs obtained on postoperative day 1 or 2.

**Table 2:** Changes in ophthalmic parameters before and after trabeculectomy.

Variable	Pre-TOM/µTLO	Pre-TLE	12 Months After TLE	*p*-Value *	*p*-Value **	*p*-Value ***
IOP (mmHg)	29.5 (26.0 to 38.0)	33.0 (28.0 to 42.0)	13.0 (11.0 to 14.0)	0.46	<0.0001	<0.0001
Medication score	5.0 (4.0 to 6.0)	6.0 (4.0 to 6.0)	0.5 (0 to 2.5)	0.03	<0.0001	<0.0001
BCVA (logMAR)	0 (−0.10 to 0.30)	0.05 (−0.02 to 0.43)	0.05 (−0.08 to 0.43)	0.02	0.83	0.02
HFA MD (dB)	−11.5 (−20.4 to −6.8)	NA	−13.2 (−21.1 to −9.5)	NA	NA	0.0049

TOM, Trabectome; µTLO, microhook trabeculotomy; TLE, trabeculectomy; IOP, intraocular pressure; BCVA, best corrected visual acuity; logMAR, logarithm of the minimum angle of resolution; HFA MD, mean deviation for Humphrey visual field. Data are presented as median (interquartile range). Wilcoxon matched-pairs signed-rank test. *p* value, * pre-TOM/µTLO vs. pre-TLE; ** pre- vs. post-TLE; *** pre-TOM/µTLO vs. post-TLE. The *p*-value was adjusted using Bonferroni correction.

**Table 3:** Postoperative outcomes and complications at 12 months after trabeculectomy.

Outcome	Study Group (n = 38)
Surgical success rate (%)	86.8
Number of failed eyes (%)	5 (13.2)
Inadequate IOP reduction	5 (13.2)
IOP < 5 mmHg	0 (0)
Loss of light perception	0 (0)
Complications of TLE	
Choroidal detachment	2
Hypotony maculopathy	0
Hyphema	1
Vitreous hemorrhage	0
Suprachoroidal hemorrhage	0
Others	IOL dislocation (1)
Needling/bleb revision (%)	10 (26.3)
Additional glaucoma surgery	2 (TLE 1, AGV 1)

Data are presented as numbers (%) unless otherwise indicated. IOP, intraocular pressure; TLE, trabeculectomy; IOL, intraocular lens; AGV, Ahmed glaucoma valve.

## Data Availability

The de-identified dataset supporting the findings of this study is available in Zenodo at https://doi.org/10.5281/zenodo.20093836, with access restricted in accordance with institutional review board requirements. Data are available from the corresponding author upon reasonable request and with appropriate ethical approval.

## Supplementary Materials

The following supporting information can be downloaded at: https://www.mdpi.com/article/10.3390/jcm15124826/s1. Supplementary Figure S1: Flow diagram of patient inclusion; Supplementary Figure S2: Kaplan–Meier analysis of surgical success stratified by the interval between MIGS and TLE; Supplementary Figure S3: Kaplan–Meier analysis of surgical success using a World Glaucoma Association–conformant definition; Supplementary Figure S4: Kaplan–Meier analysis of surgical success stratified by concomitant cataract surgery; Supplementary Figure S5: Per-eye changes in intraocular pressure between the pre-MIGS and pre-TLE periods; Supplementary Figure S6: Per-eye changes in medication burden between the pre-MIGS and pre-TLE periods.

## Abbreviations

The following abbreviations are used in this manuscript:

MIGS	Minimally invasive glaucoma surgery
TOM	Trabectome
TLO	TLO, trabeculotomy
μTLO	Microhook ab interno trabeculotomy
IOP	Intraocular pressure
TLE	Trabeculectomy
BCVA	Best corrected visual acuity
logMAR	Logarithm of the minimum angle of resolution
MD	Mean deviation for Humphrey visual field
SU-RLC	Shimane University RLC (R, red blood cells; L, layer formation; C, clots)
POAG	Primary open-angle glaucoma
SOAG	Secondary open-angle glaucoma
PXG	Pseudoexfoliative glaucoma
IOL	Intraocular lens
CRVO	Central retinal vein occlusion
SLT	Selective laser trabeculoplasty
AGV	Ahmed glaucoma valve
