# Supplementary material for: Outcomes of Salvage Trabeculectomy in Japanese Patients with Open-Angle Glaucoma and Persistent Intraocular Pressure Elevation Following Trabectome or Microhook Ab Interno Trabeculotomy
Source: J Clin Med. 2026 Jun 21;15(12):4826. doi: 10.3390/jcm15124826 (PMC13301107; doi:10.3390/jcm15124826)

Supplementary Figure S1. Flow diagram of patient inclusion.
Flow diagram showing the identification, exclusion, and inclusion of eyes in the study group and reference cohort.
TLE, trabeculectomy; TOM, trabectome; μTLO, microhook trabeculotomy; IOP, intraocular pressure.

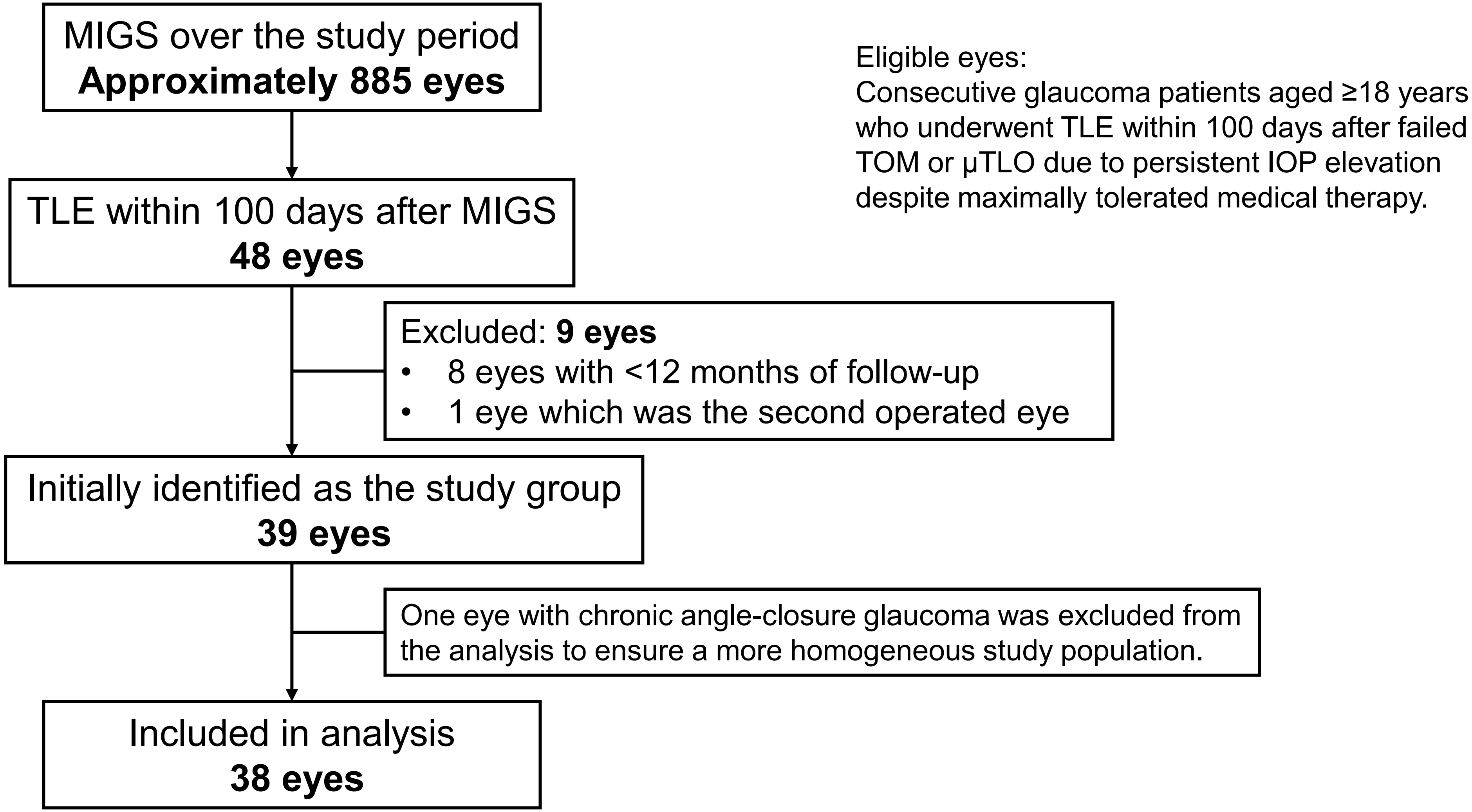

Supplement: Supplementary file 1 [file jcm-15-04826-s001.zip › S figures final/S1 Participants ver2.pdf]
