# Supplementary material for: Outcomes of Salvage Trabeculectomy in Japanese Patients with Open-Angle Glaucoma and Persistent Intraocular Pressure Elevation Following Trabectome or Microhook Ab Interno Trabeculotomy
Source: J Clin Med. 2026 Jun 21;15(12):4826. doi: 10.3390/jcm15124826 (PMC13301107; doi:10.3390/jcm15124826)
Supplement: Supplementary file 1 [file jcm-15-04826-s001.zip › S figures final/S2 interval-stratified KM final.pdf]

Supplementary Figure S2. Kaplan–Meier analysis of surgical success stratified by the interval between MIGS and TLE.

Kaplan–Meier curves comparing surgical success among eyes undergoing TLE ≤30, 31–60, and 61–100 days after MIGS. Differences were assessed using the log-rank test.

MIGS, minimally invasive glaucoma surgery; TLE, trabeculectomy.

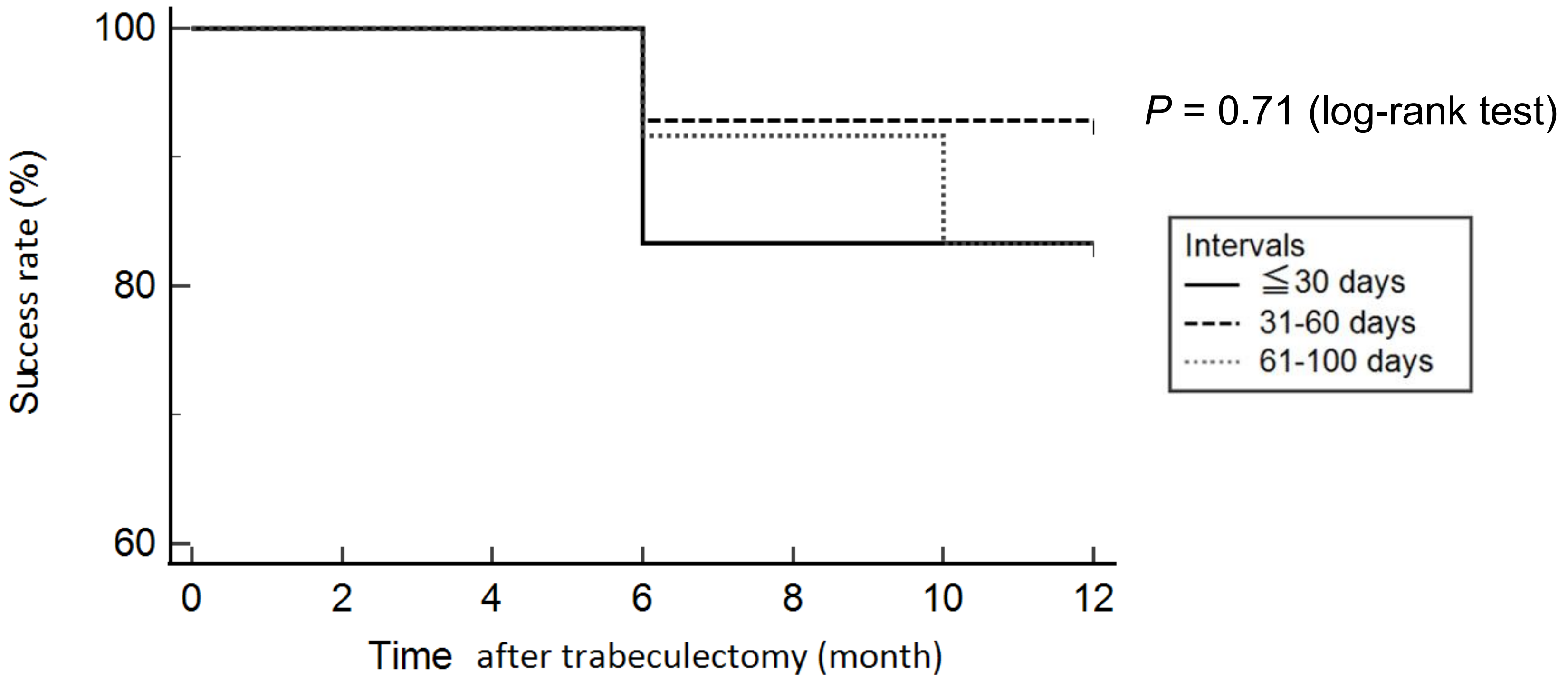

Number at risk

Group: 1							
	12	12	12	12	10	10	10
Group: 2							
	14	14	14	14	13	13	13
Group: 3							
	12	12	12	12	11	11	10
