# Supplementary material for: Outcomes of Salvage Trabeculectomy in Japanese Patients with Open-Angle Glaucoma and Persistent Intraocular Pressure Elevation Following Trabectome or Microhook Ab Interno Trabeculotomy
Source: J Clin Med. 2026 Jun 21;15(12):4826. doi: 10.3390/jcm15124826 (PMC13301107; doi:10.3390/jcm15124826)

Supplementary Figure S3. Kaplan–Meier analysis of surgical success using a World Glaucoma Association–conformant definition.
Surgical success was redefined by counting bleb needling or revision as surgical failure.

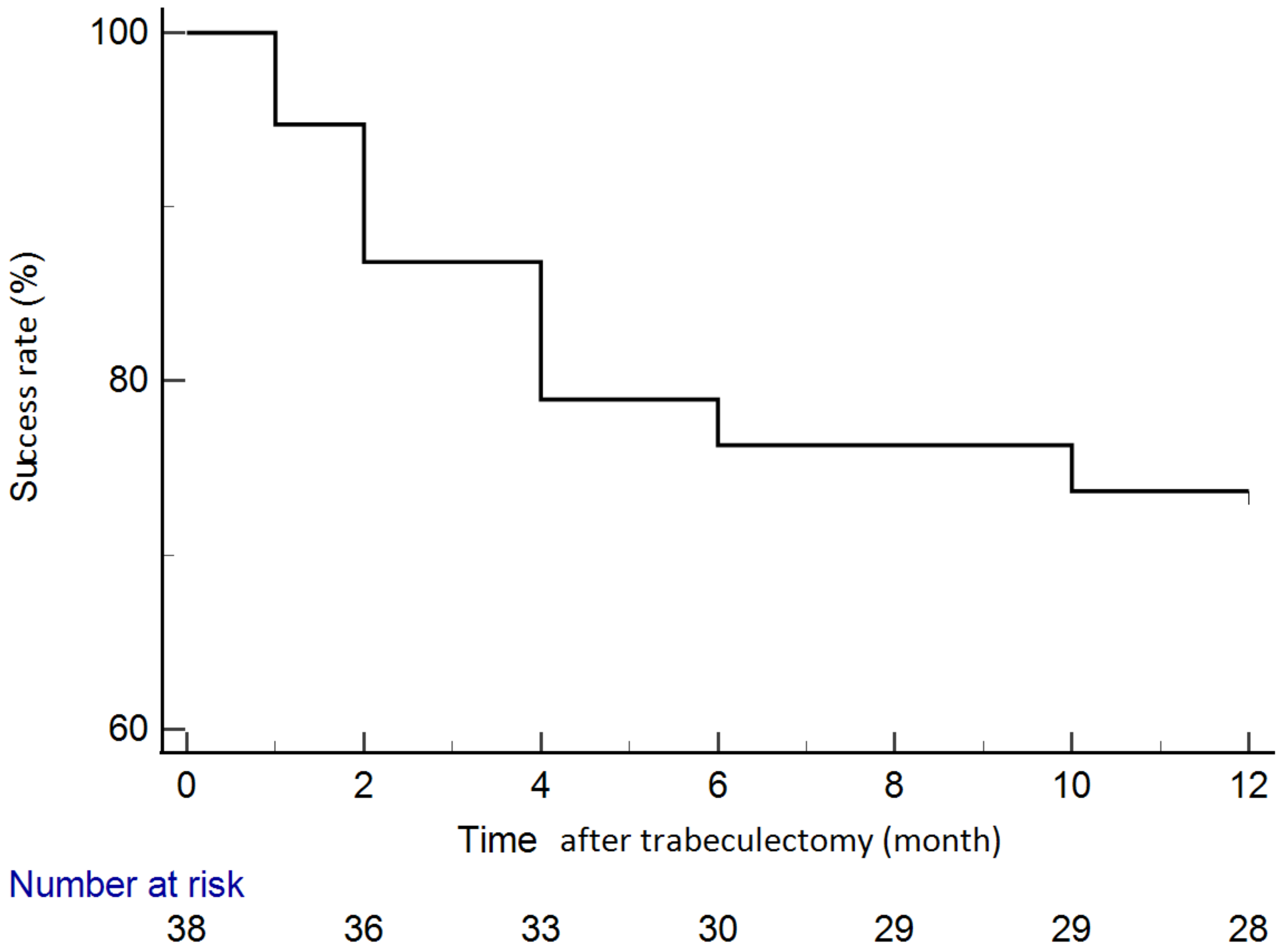

Supplement: Supplementary file 1 [file jcm-15-04826-s001.zip › S figures final/S3 KM WGA final.pdf]
