# Supplementary material for: Outcomes of Salvage Trabeculectomy in Japanese Patients with Open-Angle Glaucoma and Persistent Intraocular Pressure Elevation Following Trabectome or Microhook Ab Interno Trabeculotomy
Source: J Clin Med. 2026 Jun 21;15(12):4826. doi: 10.3390/jcm15124826 (PMC13301107; doi:10.3390/jcm15124826)

Supplementary Figure S4. Kaplan–Meier analysis of surgical success stratified by concomitant cataract surgery.

Kaplan–Meier curves comparing surgical success between eyes with and without concurrent cataract surgery at the time of MIGS. Differences were assessed using the log-rank test.

MIGS, minimally invasive glaucoma surgery.

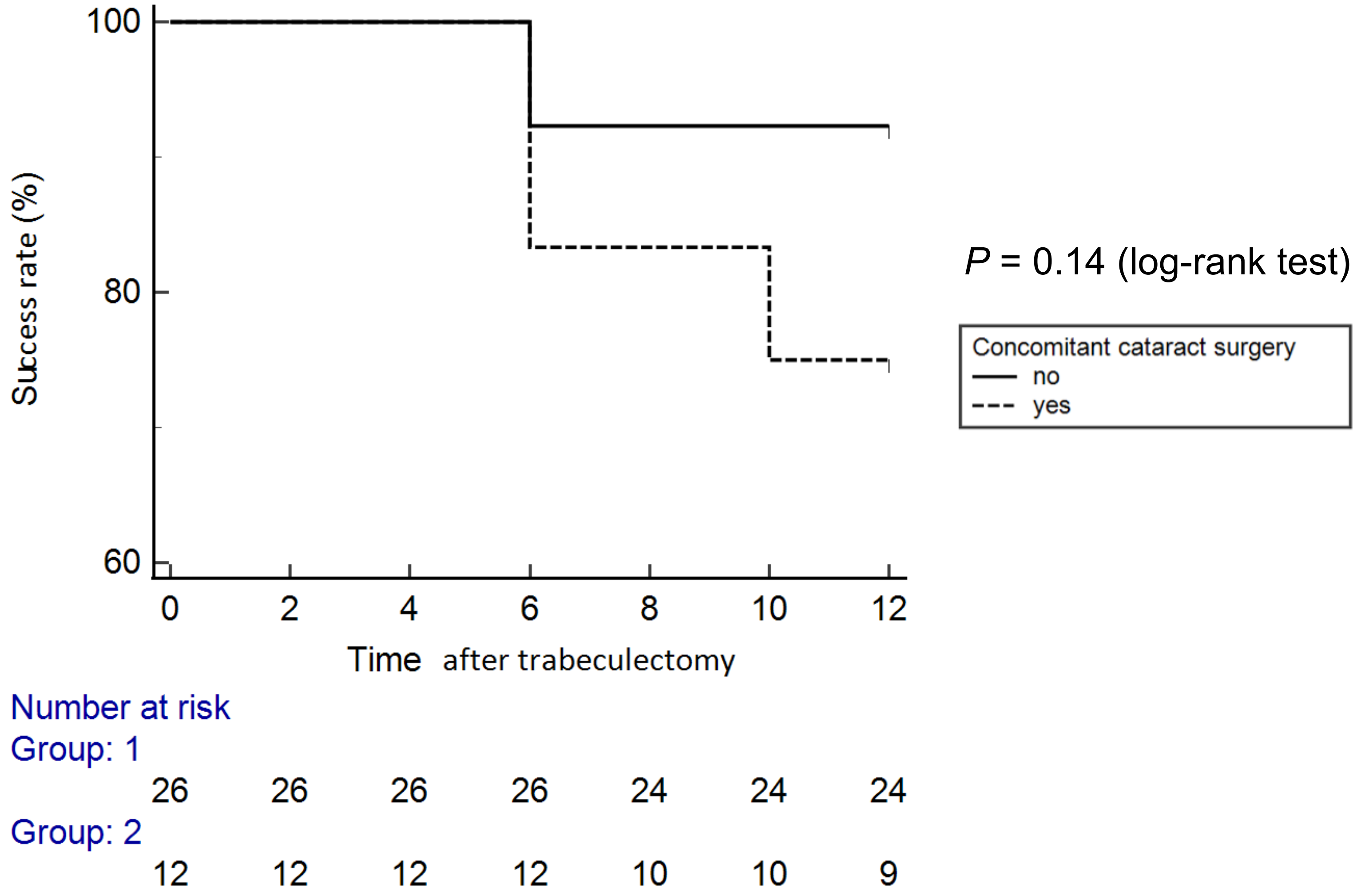

Supplement: Supplementary file 1 [file jcm-15-04826-s001.zip › S figures final/S4 cataract final.pdf]
