# Supplementary material for: Outcomes of Salvage Trabeculectomy in Japanese Patients with Open-Angle Glaucoma and Persistent Intraocular Pressure Elevation Following Trabectome or Microhook Ab Interno Trabeculotomy
Source: J Clin Med. 2026 Jun 21;15(12):4826. doi: 10.3390/jcm15124826 (PMC13301107; doi:10.3390/jcm15124826)

Supplementary Figure S5. Per-eye changes in intraocular pressure between the pre-MIGS and pre-TLE periods.

Per-eye line plots showing intraocular pressure (IOP) at the pre-MIGS and pre-TLE time points. Each line represents an individual eye, illustrating persistent elevation of IOP between the two periods prior to trabeculectomy.

IOP, intraocular pressure; MIGS, minimally invasive glaucoma surgery; TLE, trabeculectomy.

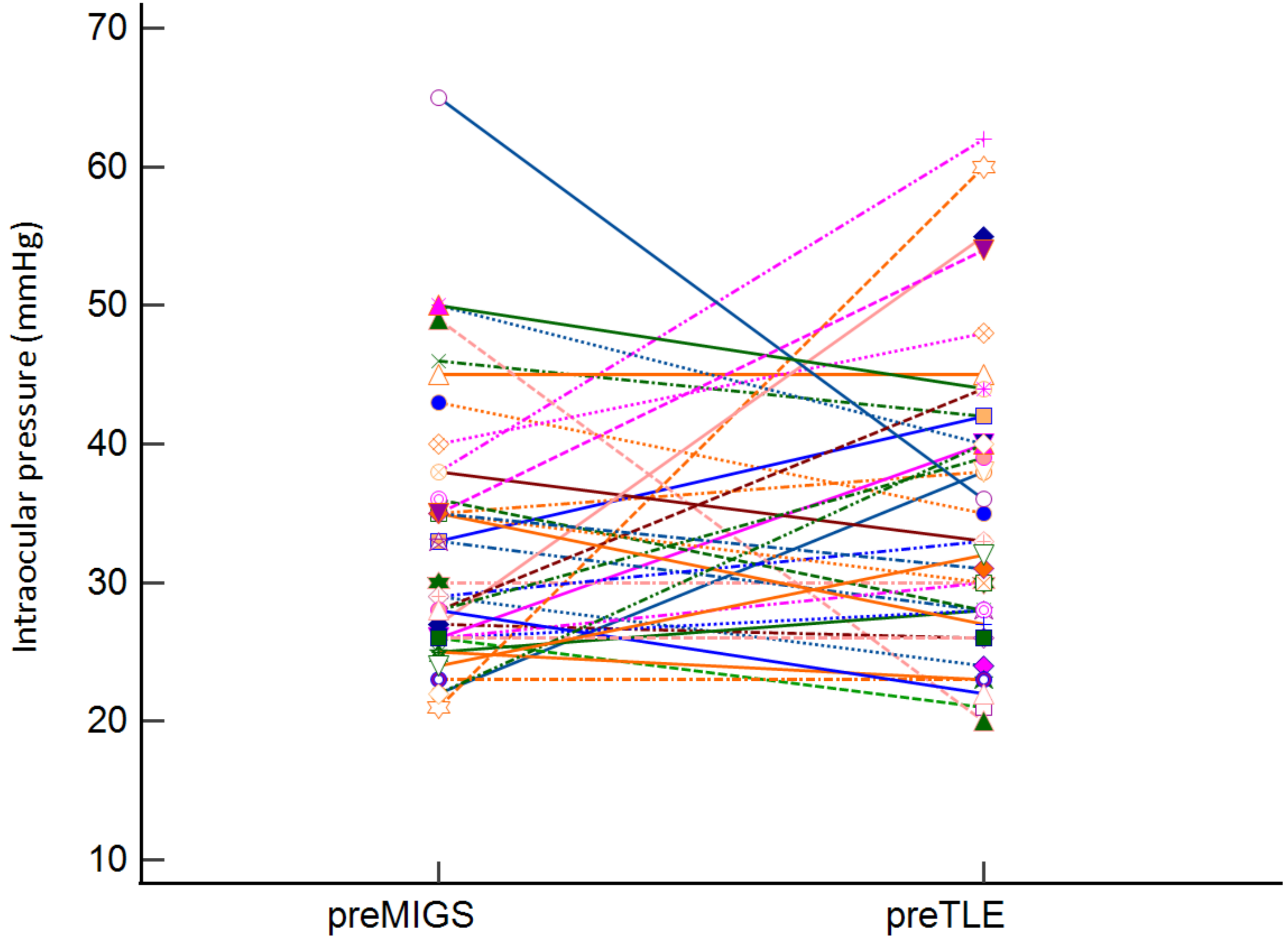

Supplement: Supplementary file 1 [file jcm-15-04826-s001.zip › S figures final/S5 IOP preMIGS and preTLE final.pdf]
