# Supplementary material for: Outcomes of Salvage Trabeculectomy in Japanese Patients with Open-Angle Glaucoma and Persistent Intraocular Pressure Elevation Following Trabectome or Microhook Ab Interno Trabeculotomy
Source: J Clin Med. 2026 Jun 21;15(12):4826. doi: 10.3390/jcm15124826 (PMC13301107; doi:10.3390/jcm15124826)

Supplementary Figure S6. Per-eye changes in medication burden between the pre-MIGS and pre-TLE periods.

Per-eye line plots showing the number of glaucoma medications used at the pre-MIGS and pre-TLE time points. Each line represents an individual eye, demonstrating an overall increase in medication burden prior to trabeculectomy.

MIGS, minimally invasive glaucoma surgery; TLE, trabeculectomy.

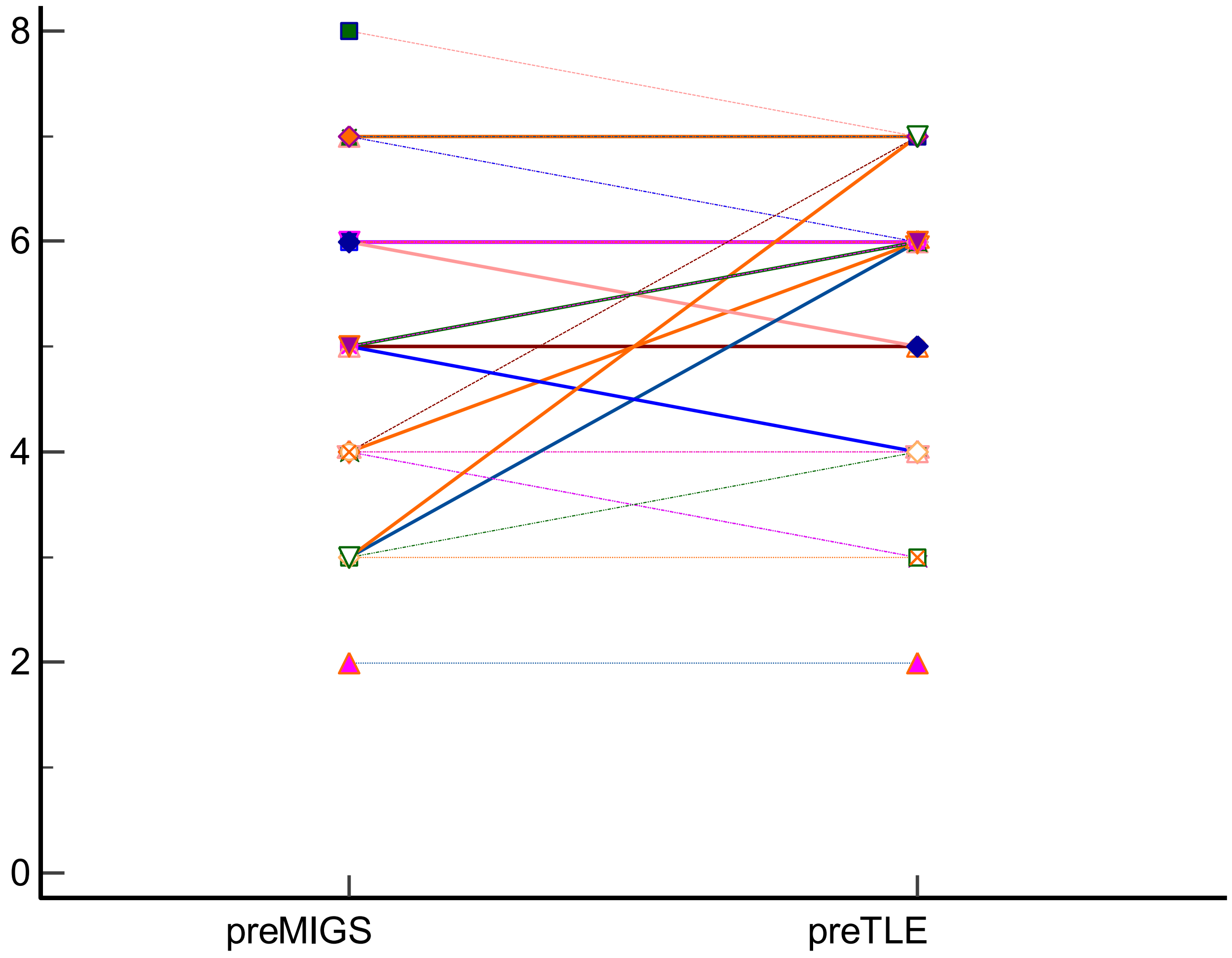

Supplement: Supplementary file 1 [file jcm-15-04826-s001.zip › S figures final/S6 Meds preMIGS and preTLE final.pdf]
